# Real-Time Visualization of the Infection and Replication of a Mouse-Lethal Recombinant H9N2 Avian Influenza Virus

**DOI:** 10.3389/fvets.2022.849178

**Published:** 2022-02-24

**Authors:** Guangjie Lao, Kaixiong Ma, Ziwen Qiu, Wenbao Qi, Ming Liao, Huanan Li

**Affiliations:** ^1^National Avian Influenza Para-Reference Laboratory (Guangzhou), South China Agricultural University, Guangzhou, China; ^2^Key Laboratory of Zoonosis, Ministry of Agriculture and Rural Affairs, Guangzhou, China; ^3^National and Regional Joint Engineering Laboratory for Medicament of Zoonoses Prevention and Control, Guangzhou, China; ^4^Key Laboratory of Zoonosis Prevention and Control of Guangdong Province, Guangzhou, China

**Keywords:** *in vivo* imaging, H9N2 AIV, 627, reporter virus, infection dynamics

## Abstract

H9N2 avian influenza viruses (AIVs) continuously cross the species barrier to infect mammalians and are repeatedly transmitted to humans, posing a significant threat to public health. Importantly, some H9N2 AIVs were found to cause lethal infection in mice, but little is known about the viral infection dynamics *in vivo*. To analyze the real-time infection dynamics, we described the generation of a mouse-lethal recombinant H9N2 AIV, an influenza reporter virus (V_K627_-NanoLuc virus) carrying a NanoLuc gene in the non-structural (NS) segment, which was available for *in vivo* imaging. Although attenuated for replication in MDCK cells, V_K627_-NanoLuc virus showed similar pathogenicity and replicative capacity in mice to its parental virus. Bioluminescent imaging of the V_K627_-NanoLuc virus permitted successive observations of viral infection and replication in infected mice, even following the viral clearance of a sublethal infection. Moreover, V_K627_-NanoLuc virus was severely restricted by the K627E mutation in PB2, as infected mice showed little weight loss and a low level of bioluminescence. In summary, we have preliminarily established a visualized tool that enables real-time observation of the infection and replication dynamics of H9N2 AIV in mice, which contributes to further understanding the mechanisms underlying the pathogenic enhancement of H9N2 AIV to mice.

## Introduction

H9N2 avian influenza virus (AIV), one of the primary subtypes of influenza virus circulating in poultry, has been widely distributed around the world (1–4). Since the H9N2 AIV was first isolated from turkeys in the US in 1966, it has been rapidly transmitted in all continents (5–7). In Asia, the H9N2 AIVs have become endemic in poultry in different countries, posing a huge economic loss to the poultry farming industry (8–12). Furthermore, H9N2 AIVs have conduced to some zoonotic events by providing the internal segments to reassortment viruses such as H7N9, H5N6, and H10N8 (13, 14). Strikingly, it has been reported that H9N2 AIVs continuously cross the species barriers to infect mammalians, including dogs, pigs, and humans (15–17). As of December 17, 2021, 93 cases of human infection with H9N2 influenza viruses have been reported worldwide, since the first human infection case was confirmed in 1998 (18, 19). Previous studies have shown that the H9N2 AIVs generally caused mild infections in poultry, even in humans. Notably, some H9N2 AIVs were found to replicate efficiently and possess lethality in mice (20–22), but the viral infection dynamics *in vivo* and the detailed mechanisms of increased pathogenicity of H9N2 AIVs to mice remain unclear, posing a potential threat to public health.

Currently, multiple influenza reporter viruses with different characteristics have been successfully used for *in vitro* or *in vivo* studies, such as the screening of antiviral compounds or neutralizing antibodies, the development of vaccines, or the identification of host factors (23–26). One of the common methods for generating a valid influenza reporter virus is to insert a reporter gene into the middle of the NS segment because it is the minimal genome of the influenza virus and this strategy can prevent any adverse effect on viral packaging signals (24, 25, 27, 28). Furthermore, different reporter genes serve different purposes, and the choice of the best reporter gene depends on the type of study. For instance, a fluorescent gene like GFP is most useful to observe localization in cells (24, 26), but luciferase is more valuable for quantitative purposes (29, 30). For *in vivo* imaging, luciferase reporters are preferred over fluorescent proteins because the sensitivity and specificity of fluorescent imaging are regularly interfered with by tissue auto fluorescence, resulting in a large amount of background (31). To analyze the viral infection dynamics in mice, the NanoLuc luciferase that possesses ~150-fold greater activity than either firefly (*Photinus pyralis*) or *Renilla luciferases* (32) was selected to generate our reporter virus.

Based on the strategy of the NS segment generating a reporter virus, we produced a recombinant H9N2 AIV expressing the NanoLuc luciferase (V_K627_-NanoLuc virus) by reverse genetics. V_K627_-NanoLuc virus possessed similar virulence and replicative capacity in mice to its parental virus. Bioluminescent imaging of the V_K627_-NanoLuc virus allowed continuous observations of viral dynamics in infected mice. Overall, these results provide an effective tool to study the real-time infection dynamics of H9N2 AIV in mice, facilitating the exploration of the mechanisms of enhanced virulence of H9N2 AIV to mice.

## Materials and Methods

### Plasmids and Cells

The eight-plasmid reverse genetics system for H9N2 avian influenza virus A/Chicken/Guangdong/V/2008 (V_K627_) was previously described (21). The non-structural (NS) gene of the influenza A virus encodes an mRNA transcript that is alternatively spliced to express two viral proteins, the non-structural protein 1 (NS1) and the nuclear export protein (NEP). The method to generate the NS1-NanoLuc-NEP segment referred to the previously reported study (27). Briefly, the NanoLuc coding sequence was placed downstream of the NS1 coding sequence via a GSGG linker, followed by a sequence of 2A peptide from porcine teschovirus (PTV-1 2A) and the NEP coding sequence. Furthermore, silent mutations were introduced into the endogenous splice acceptor site of the NS1 ORF to prevent splicing (33). The NS-NanoLuc-NEP segment maintained the non-coding sequence of the NS segment at both ends. Finally, the complete NS1-NanoLuc-NEP segment was cloned into plasmids pHW2000 with *Bsm*BI restriction sites. Madin-Darby canine kidney (MDCK) and Human embryonic kidney cells (HEK293T) were maintained in Dulbecco's modified essential medium (DMEM) with 10% fetal bovine serum (FBS) at 37°C in 5% CO_2_.

### Generation of Reassortant Viruses

V_K627_-NanoLuc virus was rescued by reverse genetics techniques (34). Briefly, 500 ng of each plasmid encoding the seven gene segments of V_K627_ and the NS-NanoLuc-NEP segment were transfected into HEK293T cells in six-well plates by using Lipofectamine 2000 (Invitrogen). After 6 hours, the medium was replaced with Opti-MEM (Gibco) including 1 μg/ml L-1-Tosylamide-2-phenylethyl chloromethyl ketone (TPCK) treated trypsin (Sigma). 48 h later, the HEK293T cells were resuspended and harvested in the medium, and the mix was injected into specific pathogen-free (SPF) chicken embryonated eggs. Then V_K627_-NanoLuc virus was confirmed by performing a hemagglutination assay. The V_K627E_-NanoLuc virus was rescued by a single K627E mutation in the PB2 protein of V_K627_-NanoLuc virus with the same protocol. V_K627_ parental virus was as a control, which contained PB2 residue K627 and was lethal to mice in our previous study (21).

### RNA Extraction, RT-PCR, and DNA Sequencing

The rescued recombinant virus (V_K627_-NanoLuc virus) was labeled as P0 and then passaged in SPF embryonated eggs for three generations (passage 1–3, P1–P3). RNA obtained from viral stocks (P0-P3) was extracted with the RNeasy minikit (Qiagen) as directed by the manufacturer. The sequences of the NanoLuc gene in the viral stocks were confirmed by reverse transcription-PCR (RT-PCR) reported by Hoffmann et al. (35) and sequencing. The specific primers used in this study were as follows (5′-3′): NanoLuc gene (Forward: ATGGTCTTCACACTCGAA; Reverse: CGCCAGAATGCGTTCGC).

### Western Blot Analysis

MDCK cells grown in six-well plates were infected with V_K627_-NanoLuc virus at a MOI of 1, and cells were lysed using RIPA Lysis Buffer (Beyotime) at 24 hours post-infection (hpi). The protein samples were followed to SDS/PAGE and transferred to a nitrocellulose membrane. NS1 protein and NS1-NanoLuc fusion protein were detected by immunoblotting with a mouse polyclonal anti-NS1 antibody (GeneTex, dilution 1:1,000) and the viral nucleoprotein (NP) was detected by immunoblotting with anti-NP antibody (SinoBiological, dilution 1:1,000), and followed by IRDye 800CW, goat anti-mouse IgG (LC-COR, dilution 1:10,000). The membrane was imaged using an Odyssey infrared imaging system (Li-CoR, United States).

### Virus Growth Kinetics

MDCK cells grown in six-well plates were infected at a multiplicity of infection (MOI) of 0.001 at 37°C, as described previously (36). One hour later, cells were washed with PBS, and then incubated with DMEM containing 1 μg/ml TPCK trypsin at 37°C with 5% CO_2_. Culture supernatants were collected at 12, 24, 36, and 48 hpi. Titers were determined by performing 50% tissue culture infective dose (TCID_50_) assays on MDCK cells.

### Mice Experiments

Pathogenicity of Viruses: 5-week-old female BALB/c mice (*n* = 5/group) (Guangdong Medical Lab Animal Center) were anesthetized with isoflurane and inoculated intranasally with V_K627E_-NanoLuc and V_K627_-NanoLuc virus at a dose of 10^6^ EID_50_/50 μl. The body weight and survival of mice were monitored daily for 14 days. Mice were humanely euthanized when they lost more than 25% of their body weight.

Determination of LD_50_: 5-week-old female BALB/c mice (*n* = 5/group) were anesthetized with isoflurane and inoculated intranasally with V_K627_ virus and V_K627_-NanoLuc virus at doses of 10^3^, 10^4^, 10^5^, or 10^6^ EID_50_/50 μl. The body weight and survival of mice were monitored daily over a period of 14 days. Mice were humanely euthanized when they lost more than 25% of their body weight. LD_50_ values were calculated by the method of Reed and Muench. To detect the viral replication in the lungs of mice, another three mice from the group of 10^6^ EID_50_/50 μl were euthanized at 5 days post-infection (dpi) and viral titers in the lungs were determined by an EID_50_ assay.

#### *In vivo* Imaging

The same mouse from each group was imagined at the appointed time by using an IVIS imaging system. Briefly, infected mice were anesthetized with isoflurane and 100 μl Nano-Glo reagent (Promega, dilution 1:25) was injected into retro-orbitally of mice. The Living Image software was used for image acquisition and analysis. Flux measurements were automatically calculated from the signal of mice. All data of composite images used the same scale.

### Statistics

Data represent means ± standard deviations (SD) (*n* ≥ 3) unless otherwise noted. Multiple comparisons were performed by using an unpaired *t*-test in the GraphPad Prism software (GraphPad Software Inc.). Significance is defined as *P* < 0.05 and is indicated with an asterisk (^*^).

## Results

### Generation of a Mouse-Lethal Influenza Reporter Virus

**Figure 1:**
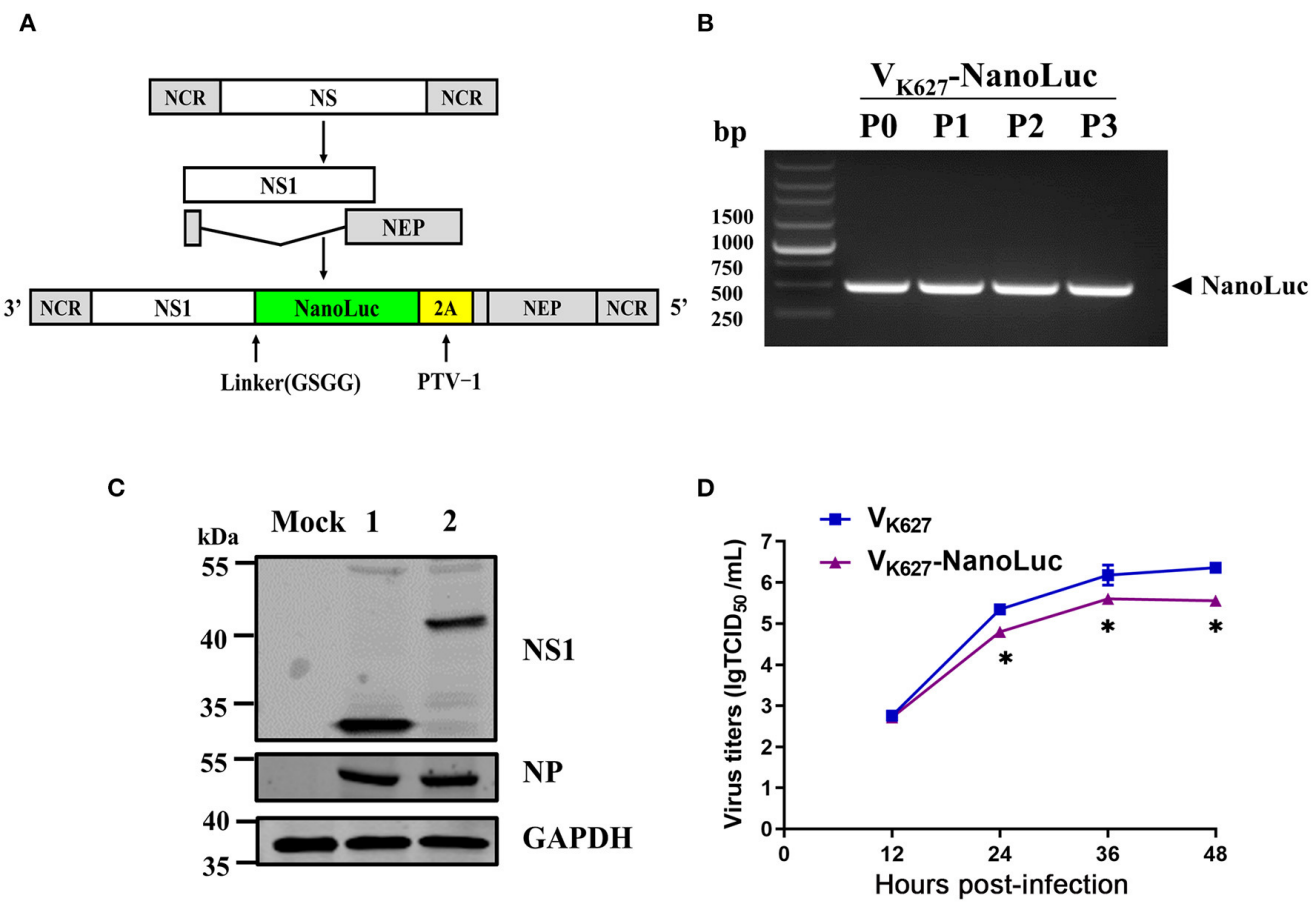
Generation of an influenza reporter virus carrying a NanoLuc gene. **(A)** Schematic diagram of NS segments of V_K627_ virus and V_K627_-NanoLuc virus. NS1 coding sequence (white) was fused to NanoLuc coding sequence (green) via a GSGG linker, followed by a sequence of 2A peptide from porcine teschovirus (PTV-1 2A, yellow) and the NEP coding sequence (gray). **(B)** The rescued V_K627_-NanoLuc virus was labeled as P0 and then passaged three times in SPF embryonated eggs (P1–P3). The NanoLuc gene of viral stocks (P0–P3) was detected by RT-PCR. **(C)** MDCK cells were infected with parental virus V_K627_ (lane 1) and V_K627_-NanoLuc virus (lane 2) at a MOI of 1. At 24 hours post-infection (hpi), NS1, and NP proteins were detected by Western blot analyses. **(D)** MDCK cells were infected with V_K627_ virus and V_K627_-NanoLuc virus at a multiplicity of infection (MOI) of 0.001. Culture supernatants collected at 12, 24, 36, and 48 hpi were determined by TCID_50_ assays on MDCK cells. Data represent the means ± standard deviations (SD) of the results determined for triplicate wells. Statistical significance was determined by an unpaired *t*-test (*P < 0.05, *n* = 3).

The NS segment of the influenza virus encodes NS1 protein and NEP protein produced from unspliced mRNA and spliced mRNA, respectively (Figure 1A). It has been previously reported that NS1 and NEP can be expressed and separated effectively by the cleavage site of 2A from porcine teschovirus (PTV-1 2A) during translation (27). In this study, we altered the NS segment of the V_K627_ virus, which led to NS1-NanoLuc as a fusion protein with PTV-2A cleavage site, allowing NEP protein to be separated from the NS1-NanoLuc fusion protein during translation (Figure 1A). By using reverse genetics techniques, a recombinant H9N2 AIV encoding the NanoLuc luciferase (V_K627_-NanoLuc virus) was rescued (marked as P0). The amino acid at position 627 of the PB2 protein on both V_K627_-NanoLuc virus and parental virus V_K627_ is lysine (K).

The stability of the NanoLuc reporter gene was tested following amplification in SPF embryonated eggs. The rescued V_K627_-NanoLuc virus (P0) was propagated in SPF embryonated eggs for three generations (passages 1 to 3, P1-P3). RT-PCR and Sanger sequencing were performed on RNA obtained from viral stocks. As shown in Figure 1B, the NanoLuc gene was detectable using specific primers in all the V_K627_-NanoLuc viruses. To detect the expression of NS1-NanoLuc fusion protein, Western blot analysis was performed in MDCK cells (Figure 1C). In the V_K627_ virus-infected cells, NS1 protein (~ 25 kDa) was detected. In V_K627_-NanoLuc virus-infected cells, the band corresponding to NS1-NanoLuc fusion protein (~ 43 kDa) was observed. These results showed that the V_K627_-NanoLuc virus expressed NS1 protein and NanoLuc luciferase as a fusion protein.

To test whether the presence of a longer NS1-NanoLuc-NEP segment in V_K627_- NanoLuc virus affected the viral replication in culture, we compared the growth kinetics of V_K627_-NanoLuc virus and parental virus V_K627_ in MDCK cells (Figure 1D). MDCK cells were infected with V_K627_-NanoLuc and V_K627_ virus at a MOI of 0.001, and the viral titers in the supernatant at various hpi were determined by TCID_50_ assay. Compared with the V_K627_ virus, V_K627_-NanoLuc virus showed attenuated replication in MDCK cells, with maximum titers reaching up to 5.60 lgTCID_50_/Ml. This indicated that V_K627_-NanoLuc virus replicated efficiently in MDCK cells, though reaching about 10-fold lower titers than the parental virus.

### V_K627_-NanoLuc Virus Causes Severe Pathogenicity in Mice

**Figure 2:**
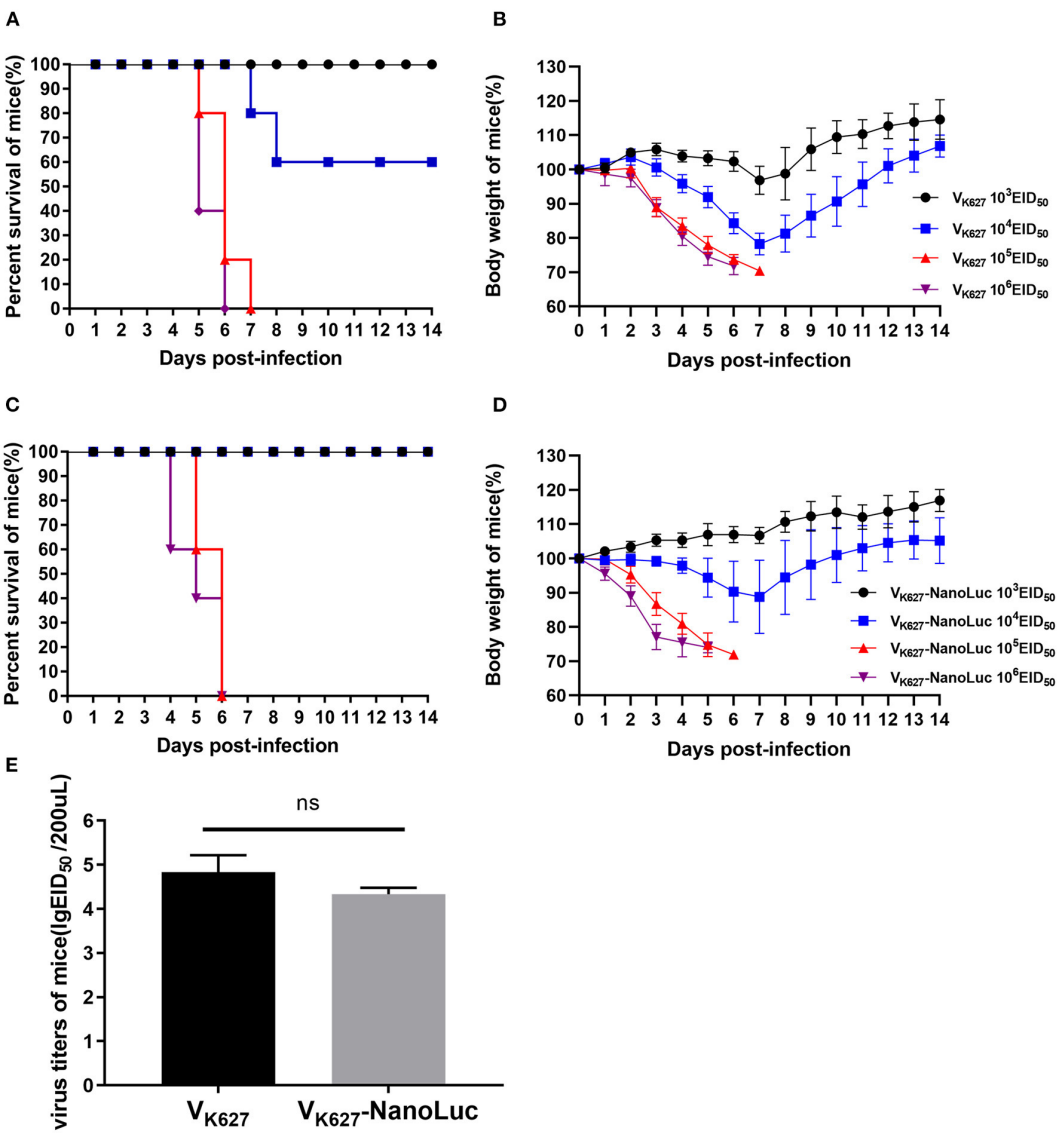
The pathogenicity of V_K627_-NanoLuc virus *in vivo*. BALB/c mice (*n* = 5) were infected intranasally with 10^3^, 10^4^, 10^5^, or 10^6^ EID_50_/50 μl of V_K627_ virus. Survival **(A)** and body weight **(B)** were monitored for 14 days. BALB/c mice (*n* = 5) were infected intranasally with 10^3^, 10^4^, 10^5^, or 10^6^ EID_50_/50 μl of V_K627_-NanoLuc virus. Survival **(C)** and body weight **(D)** were monitored for 14 days. **(E)** Virus titers in the lungs of infected mice with 10^6^ EID_50_/50 μl at 5 days post-infection (dpi). The data are shown as means ± standard deviations (SD) (*n* = 3). Statistical significance was determined by an unpaired *t*-test (*P < 0.05, ns: no significant difference).

V_K627_ virus caused significant pathogenicity in mice as previously described in our study (21, 37). To test whether the V_K627_-NanoLuc virus was comparable pathogenicity to its parental virus, five mice of groups were infected with 10^3^, 10^4^, 10^5^, or 10^6^ EID_50_/50 μl of V_K627_ virus and V_K627_-NanoLuc virus, monitored daily for survival and weight loss. We found that survival and weight loss showed an intense dose-dependent effect (Figures 2A–D). In the parental virus-infected mice, all mice inoculated with 10^5^ EID_50_/50 μl or higher and a minority of those infected with 10^4^ EID_50_/50 μl succumbed to infection (Figures 2A,B). In the V_K627_-NanoLuc virus-infected mice, all mice inoculated with 10^5^ EID_50_/50 μl or higher succumbed to infection, whereas all mice infected with 10^4^ EID_50_/50 μl survived (Figures 2C,D). According to the survival data, the LD_50_ value of V_K627_-NanoLuc virus was determined as 10^4.5^ EID_50_/50 μl, only 2.3-fold higher than those of the parental virus (10^4.13^ EID_50_/50 μl).

We determined the viral titers in the lungs of three mice infected with 10^6^ EID_50_/50 μl at 5 dpi. The result showed that the V_K627_-NanoLuc virus replicated efficiently in the lungs of mice, with mean titers reaching 4.33 lgEID_50_/200 μl, slightly lower than V_K627_ virus (4.83 lgEID_50_/200 μl) (Figure 2E). Combined, these data indicated that V_K627_-NanoLuc virus replicated to high levels and caused significant pathogenicity in mice, which was close to those of the parental virus.

### Real-Time *in vivo* Imaging of V_K627_-NanoLuc Virus Infection

**Figure 3:**
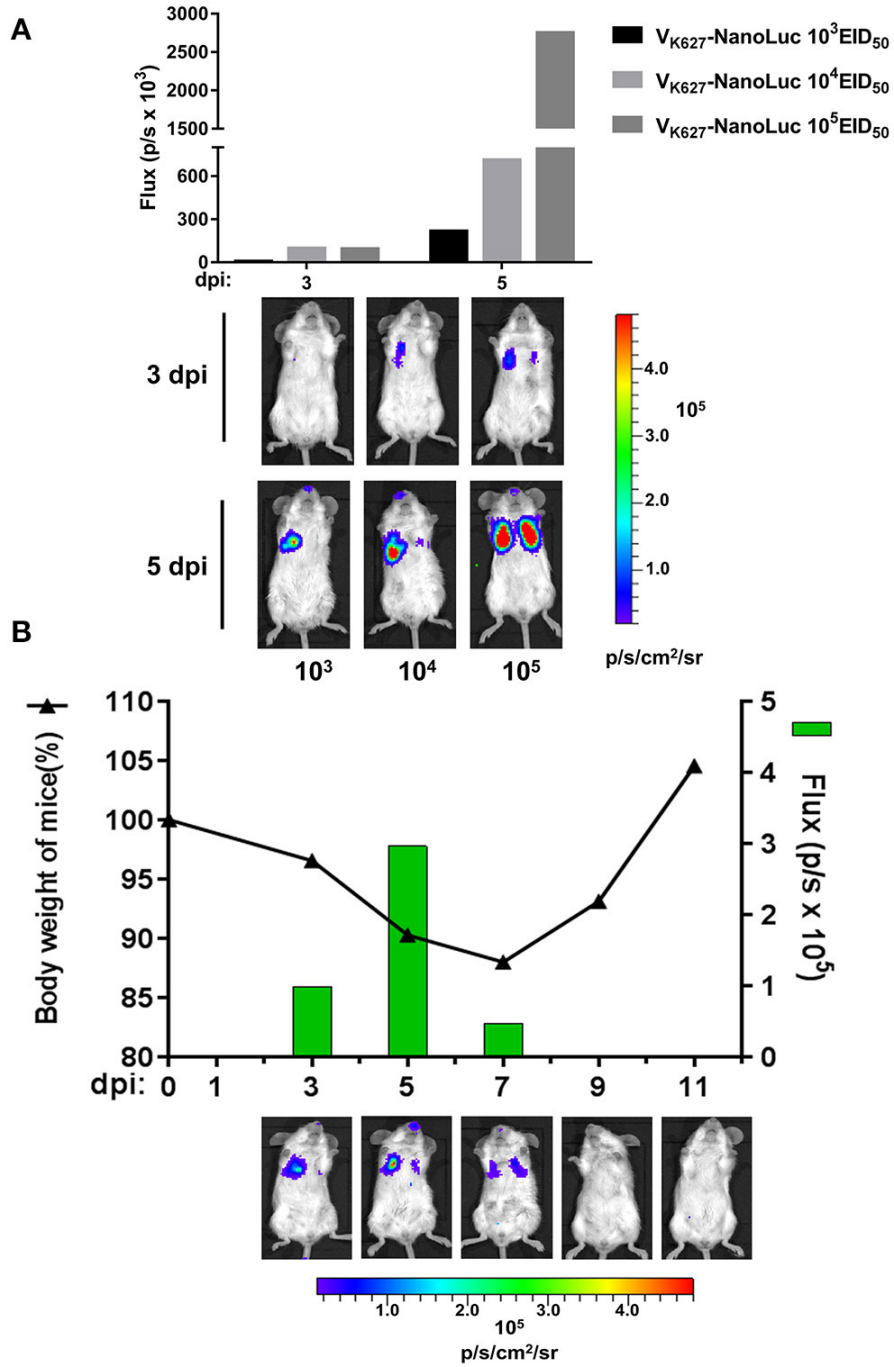
*In vivo* imaging of V_K627_-NanoLuc virus. **(A)** Mice were infected intranasally with V_K627_-NanoLuc virus at doses of 10^3^, 10^4^, and 10^5^ EID_50_/50 μl. The correlation between bioluminescent signal and the amount of viral inoculum was examined by longitudinal observation of infected mice. Representative data from serial imaging of one mouse are shown. **(B)** Mice infected with 10^4^ EID_50_/50 μl of V_K627_-NanoLuc virus and serial imaging of the same single mouse was performed. The relationship between weight loss and bioluminescence signal was shown. All data of composite images used the same scale.

It has been proven that *in vivo* imaging of reporter viruses is useful to study the viral infection dynamics for different viruses, including influenza A virus (38, 39), dengue virus (40, 41), and vaccinia virus (42). To analyze the real-time infection dynamics of a mouse-lethal H9N2 AIV, V_K627_-NanoLuc virus was used to visualize the replication. We performed infections with mice at indicated doses of V_K627_-NanoLuc virus and the same mouse from each group was imagined at 3 and 5 dpi by using an IVIS imaging system. Longitudinal imaging of the same infected mouse from each group showed that the bioluminescent signal from V_K627_-NanoLuc virus-infected lungs increased as the dose of the infection increased (Figure 3A). To better explore the viral infection dynamics, a single mouse was infected with the V_K627_-NanoLuc virus at a sublethal dose of 10^4^ EID_50_/50 μl. Then we imagined the infected mouse and monitored its body weight at 3, 5, 7, 9, and 11 dpi, respectively. The correlation could be observed between the body weight and the bioluminescent signal from the same mouse infected with the V_K627_-NanoLuc virus (Figure 3B). The bioluminescent signal was mainly detected in the left lung accompanied by weight loss in the mouse at 3 dpi. The bioluminescent signal peaked and displayed on both sides at 5 dpi, suggesting the spread of the influenza virus to the right lung. Bioluminescent intensity decreased at 7 dpi, suggesting the viral infection began to decline. The bioluminescent signal was undetectable at 9 dpi, accompanied by a rise in body weight, indicating the clearance of infection was continued. In brief, the V_K627_-NanoLuc virus was capable of permitting serial observation of viral replication and clearance in real time.

**Figure 4:**
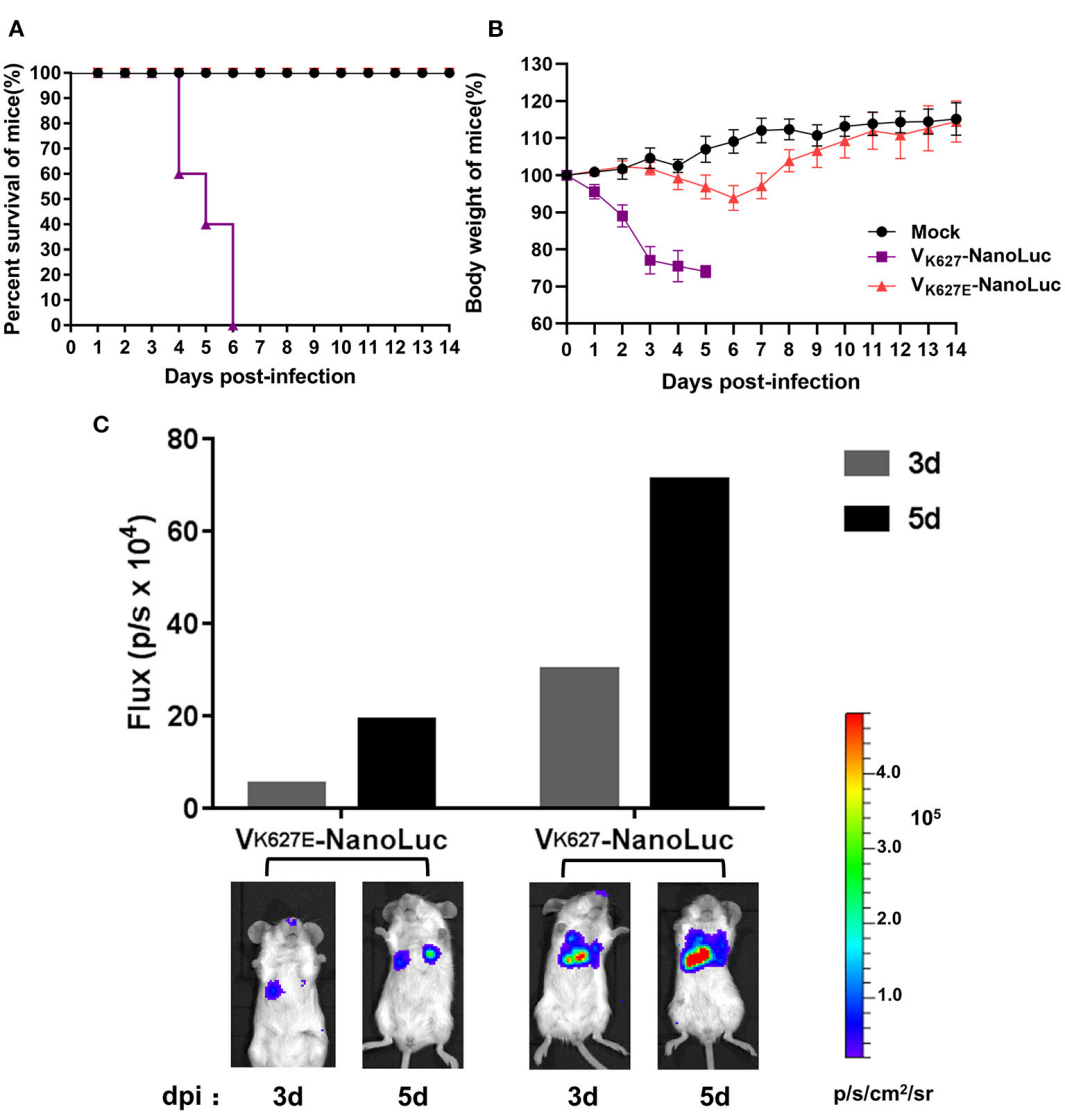
V_K627_-NanoLuc virus was attenuated in mice by a single K627E mutation in the PB2 protein. Mice (*n* = 5) were infected with 10^6^ EID_50_/50 μl of V_K627_-NanoLuc virus and V_K627E_-NanoLuc virus. Survival **(A)** and body weights **(B)** were monitored for 14 days. **(C)** Imaging of the same single mouse of each group was performed at 3 and 5 dpi.

More evidence has demonstrated that PB2 residue K627 is a critical factor of virulence and host range for influenza A viruses, which can significantly facilitate the adaptability of viruses and contribute to enhanced virulence in mice (43, 44). In this study, our reporter virus harboring PB2 residue K627 causes high pathogenicity in mice, showing similar results to those of the previous study. To test whether the K627E mutation in PB2 protein had an impact on the virulence of our reporter virus, the V_K627E_-NanoLuc virus was rescued by a single K627E mutation in the PB2 protein of the V_K627_-NanoLuc virus. We performed infections in mice with 10^6^ EID_50_/50 μl of V_K627E_-NanoLuc virus and V_K627_-NanoLuc virus, monitoring daily for survival and weight loss, and imagined the infected mice at 3 and 5 dpi by an IVIS imaging system. As expected, mice infected with V_K627_-NanoLuc virus lost weight and succumbed to infection, displaying a strong bioluminescence signal. In contrast, mice infected with V_K627E_-NanoLuc lost little weight and survived, showing a low level of bioluminescence signal (Figure 4).

## Discussion

H9N2 AIVs have been distributed in various avian species worldwide and are repeatedly spread to mammals. In general, H9N2 infections in poultry or human are mild. However, some H9N2 AIVs were reported to cause lethal infection in mice (20–22). To better understand the mechanisms, we generated a mouse-lethal recombinant H9N2 AIV (V_K627_-NanoLuc virus) to analyze the real-time infection dynamic of H9N2 AIV in mice. Our work has demonstrated that the virulence and replicative capacity of the V_K627_-NanoLuc virus in mice were very close to its parental virus. Furthermore, *in vivo* imaging of our reporter virus was able to visualize viral replication in mice models.

The creation of replication-competent influenza reporter viruses is interfered with by the complex structure of segmented genomes (26). All viral genes are essential *in vivo*, so large insertions or a simple replacement of reporter genes may severely attenuate viral replication. Most importantly, the inappropriate location of the reporter gene insertion can interfere with viral packaging signals and subsequently affect virus assembly. In our reporter virus, a small NanoLuc reporter gene was inserted into the middle of the NS segment to circumvents the repetition of packing signals. In the same strategy, various recombinant influenza viruses carrying foreign genes in their NS segments have been successfully used *in vitro* and *in vivo* (24, 27, 28). Although V_K627_-NanoLuc virus was attenuated for replication in MDCK cells, it replicated effectively in the lungs and showed near-native pathogenicity properties in mice. These improvements allowed us to monitor the infected mice *in vivo* during infection with our reporter virus.

More evidence has demonstrated that PB2 protein 627 plays a key role in virulence and host range for influenza A viruses, facilitating the adaptability of viruses in mammals (8, 43, 44). It's reported that the higher pathogenicity of H9N2 viruses in mice is associated with the PB2 E627K mutation (21, 45). Adaptation of H9N2 AIV to mice results in multiple amino acid substitutions, including E627K in PB2 protein (43). Our previous study indicated that the PB2 E627K mutation of H9N2 AIV contributed to enhanced virulence in mice by inducing a higher level of glucocorticoids (GCs) (46). However, the detailed mechanisms that increase the pathogenicity of H9N2 AIV in mice are still poorly understood. In this study, the characteristics of our reporter virus harboring PB2 residue K627 were consistent with those of previously reported viruses, and our reporter virus could provide a practical tool for further studies on understanding the mechanisms of increased virulence of the H9N2 AIV to mice.

In summary, we have generated an influenza reporter virus encoding the NanoLuc luciferase, which was lethal to mice and was usable for *in vivo* imaging. Our report virus was able to track the real-time dynamics of infection and replication of H9N2 AIV in mice. Besides, our reporter virus could be applied to rapidly screen and assess the efficacy of antiviral therapies and vaccines.

## Data Availability Statement

The original contributions presented in the study are included in the article/supplementary material, further inquiries can be directed to the corresponding author/s.

## Ethics Statement

The animal study was reviewed and approved by South China Agriculture University Institutional Animal Care and Use Committee.

## Author Contributions

GL, WQ, ML, and HL conceived and designed research and wrote modified the manuscript. GL, KM, ZQ, and HL conducted experiments. ML and HL revised the manuscript. All authors read and approved the manuscript.

## Funding

This work was supported by National Natural Science Foundation of China (32102662 and 31830097), China Postdoctoral Science Foundation Funded Project (2021M691082), and Young Scholars of Yangtze River Scholar Professor Program (2019, WQ).

## Conflict of Interest

The authors declare that the research was conducted in the absence of any commercial or financial relationships that could be construed as a potential conflict of interest.

## Publisher's Note

All claims expressed in this article are solely those of the authors and do not necessarily represent those of their affiliated organizations, or those of the publisher, the editors and the reviewers. Any product that may be evaluated in this article, or claim that may be made by its manufacturer, is not guaranteed or endorsed by the publisher.
